# The Novelty Effect as a Predictor of Language Outcome

**DOI:** 10.3389/fpsyg.2019.00258

**Published:** 2019-02-12

**Authors:** Caterina Marino, Judit Gervain

**Affiliations:** ^1^Integrative Neuroscience and Cognition Center (INCC) (UMR 8002), Université Paris Descartes, Paris, France; ^2^INCC (UMR 8002), CNRS, Paris, France

**Keywords:** infants, novelty effect, language development, cognitive abilities, predictability

## Abstract

A controversial issue in the field of language acquisition is the extent to which general attentional or cognitive abilities play a role in individual differences in early language outcomes. Here we report a longitudinal study where we examined whether processing efficiency in a novelty detection task predicted later vocabulary size in a stable manner across time. We found that the novelty detection ability measured at 9 months was significantly predictive of later vocabulary size at 12, 14, 18, and 24 months. This study, therefore, emphasizes the importance of controlling for non-linguistic factors when assessing individual variability in language development. A more accurate assessment of language development may be obtained if general attentional and cognitive abilities are also taken into account in addition to linguistic factors.

## Introduction

Acquiring language is one of the characteristics that makes human beings unique. Indeed, young learners achieve this task quite easily and effortlessly. Infants across different languages uniformly go through the same sequence of large developmental milestones (e.g., Kuhl, 2004) suggesting that language development shows a universal overall trajectory. However, within this broadly universal pattern, individual variability is present in the timing, quality, rate and efficiency of early linguistic abilities. These individual variations are more often studied with atypical participants (e.g., Rose et al., 2005), and it is often assumed that language development is homogeneous in typically developing, healthy infants. Nevertheless, a growing body of evidence has revealed individual differences in speech perception and production in typically developing infants, as well (e.g., Slobin and Bever, 1982; Newman et al., 2006; Cristia and Seidl, 2011).

After decades of research, we now have a relatively good understanding of many of the linguistic processes that are fundamental in language acquisition. Nevertheless, individual variation can sometimes be obscured by group data. Recently, an increasing number of studies have started highlighting the importance of considering individual differences between learners as a way of shedding light on underlying learning mechanisms (e.g., Newman et al., 2006; Cristia and Seidl, 2011). The investigation of individual variation might help to describe and predict individual trajectories more accurately.

Several studies have pointed out that language learning is influenced by language-external factors. General attentional mechanisms and cognitive constraints (Bloom, 1993; Hollich et al., 2000; Rose et al., 2009), socio-economic status and parental input (e.g., Fernald et al., 2013; Weisleder and Fernald, 2013) have been shown to influence infants’ linguistic outcomes.

Indeed, assessing these language external factors and taking them into account is an important challenge in language acquisition research, as they might affect infants’ performance in a laboratory environment. In studies that are aimed at assessing individual variability, taking these factors into account is even more important. However, while the involvement of general cognitive abilities in later language acquisition is a central issue in atypical development (e.g., McCall and Carriger, 1993; Rose et al., 2005), fewer studies have investigated this specific link with typical, healthy infants (e.g., Thompson et al., 1991; Rose et al., 2009). Therefore, the purpose of this longitudinal study is to examine whether a novelty detection/dishabituation task, measuring general attentional and cognitive skills, predicts vocabulary growth at later ages in the typical population. With this study, a better understanding of the basic developmental trajectories of language development might be achieved.

We focus on these general cognitive skills, as their contribution to later language outcomes is controversial in the literature. Some authors consider that general cognitive mechanisms, such as attention or memory, are not sufficiently stable during early development and thus a poor measure of individual ability at any later stage (Bayley, 1949; McCall, 1979; Kopp and McCall, 1982). Some studies have also proposed that there is huge variability in the magnitude of the cognitive effects, which makes it difficult to link them directly to later outcomes (Bayley, 1969). Other researchers, by contrast, have proposed that looking time measures, like the increased response to a novel stimulus, are good predictors of later intelligence (Berg and Sternberg, 1985; Sternberg, 1985; Bornstein and Sigman, 1986). A meta-analysis of studies with both at-risk and typically developing infants reported that measures of infants’ habituation and novelty detection performance reliably predicted later IQ assessed between 1 and 8 years of age. Nevertheless, even if the predictions were reliable for both populations, a higher degree of predictability was reported for the samples of at-risk infants (McCall and Carriger, 1993).

As visual attention is one of the major sources of infants’ knowledge of the world and constitutes a simple, observable behavior, looking time measures have largely been used to discover the cognitive mechanisms supporting infants’ cognitive and linguistic development (as measured by the CDI), (e.g., Colombo et al., 1989; Thompson et al., 1991; Rose et al., 2009). Differences in looking times reflect distinct underlying cognitive processes, which in turn may be linked to individual differences (see Aslin, 2007 for a review). In one frequently used looking time paradigm, the habituation-dishabituation or novelty detection paradigm, infants are repeatedly exposed to a stimulus until they habituate, i.e., their looking times considerably decrease, defined as reaching a predefined criterion, e.g., a 50 or 60% decrement in looking times compared to an initial baseline. If a new stimulus is presented after habituation, and looking times significantly increase, i.e., dishabituation occurs, this is interpreted as infants’ ability to detect and discriminate the novel stimulus.

Responding to novelty involves two fundamental aspects. First, there is a “motivational” aspect by showing interest in or attending to something novel. Second, there is an “information-extraction/memory” aspect that involves the ability to identify features necessary to encode novel information and compare it to older information (Berg and Sternberg, 1985). Nevertheless, the underlying mechanisms linking specific cognitive abilities and later language outcomes are just beginning to be explored. For instance, sensitivity to novelty may be hypothesized to play a crucial role in development, because it allows the infant to direct attention to information that is not yet known.

The current study has, therefore, used a visual novelty detection task as a potential predictor of language outcome. It is one of the most commonly used measures of infants’ general cognitive abilities, it is short and easy to administer and it is in a different perceptual modality (visual) than spoken language, ensuring that it taps into language-external abilities. Responding to novel information involves the ability to encode novel information and compare them with older ones. Therefore, this response has widely been used to study discrimination, memory, categorization, discrimination and concept formation in infants (e.g., Sternberg, 1985). Moreover, several studies have validated it as a predictive measure of the development of general cognitive functions during childhood (e.g., Berg and Sternberg, 1985; Bornstein and Sigman, 1986).

The visual novelty detection task has been found to predict later cognitive abilities as well as language outcome in a few existing studies. In one of the first studies, early novelty preference was found to be related to later memory skills (e.g., Colombo et al., 1989). Subsequently, Thompson et al. (1991) administered a test of visual novelty preference (FTII) to American infants at 5 and 7 months. The infants were followed longitudinally and the Bayley Scales of Infant Development (Bayley, 1969) was administered at 12 months, whereas the MacArthur-Bates Communicative Development Inventories (Fenson et al., 1993) were administered at 24 and 36 months together with the Stanford-Binet and the Colorado Specific Cognitive Abilities Test at 36 months. They found that early novelty preference was highly correlated with IQ (as measured with the Stanford-Binet test) at 24 and 36 months (not at 12 months). Furthermore, it also predicted language skills at 36 months. More recently, Rose et al. (2009) used a large battery of tasks to measure infants’ attention, memory, speed of processing and representational competences (operationalized in the study as the ability to extract commonalities from different stimuli and represent them more abstractly or in a generalized way). Authors indeed observed that several of the measures can be used as predictors of later language outcomes. Infants’ memory and representational competence were related to language at both 12 and 36 months in a concurrent and predictive way.

In a longitudinal study, Benasich and Tallal (1996, 2002) tested whether early auditory processing efficiency predicted language outcome. Specifically, the ability to process stimuli presented rapidly and sequentially, known as Rapid Auditory Processing (RAP), was tested. This ability involves the discrimination of two (or more) sounds presented one after the other, and can be operationally measured by determining the minimum interval between the two sounds that is required for successful discrimination. The RAP paradigm has been extensively used to predict subsequent language outcomes. Benasich and Tallal (1996, 2002) tested the hypothesis that difficulties in acoustic processing might impair phonemic mapping, thus delaying later language acquisition. By comparing 7-month-old infants at risk for specific language impairment (SLI) with typically developing peers, they have shown that early differences in acoustic processing are present since the earliest stages of learning (e.g., Benasich and Tallal, 1996). Moreover, they found that infants whose acoustic efficiency, as measured by the RAP, diverges from the norm also show evidence of less efficient processing and delayed language acquisition over time. Specifically, measures of the receptive and expressive vocabulary were evaluated at 12, 16, 24, and 36 months. The RAP threshold was found to be the best predictor of language outcomes at 24 months. Crucially in all the measures, infants at-risk performed worse than controls, exhibiting poorer auditory processing abilities than controls at the same age, and these poorer auditory abilities were correlated with later linguistic behavior. This is strong evidence that early deficits in RAP precede and predict language delays (Benasich and Tallal, 2002).

Relevantly for our purpose, in these studies, Benasich and Tallal (1996, 2002) also tested cognitive/attentional mechanisms using a visual novelty detection task in an attempt to understand whether the processing deficit observed in the RAP was specific to the auditory domain in the SLI cohort or was a more general cognitive processing deficit. Infants were habituated to repeated presentations of a face and then tested with a familiar versus a novel face. Atypical infants were weaker at detecting novelty than their typical peers. Moreover, several variables derived from the RAP and the novelty detection tasks were correlated. The authors interpreted this as evidence that the two tasks may be tapping onto similar processes, suggesting that information processing may not be modality specific (Benasich and Tallal, 1996).

In the light of the above, this study measured the novelty effect in French monolingual infants at 9 months of age. The results of the study show that this effect is an early predictor of later linguistic outcomes.

Our investigation had three main goals and unique contributions. First, very few of the previous studies have looked at the novelty effect as an early predictor of vocabulary in a longitudinal manner. Thus it remains unknown whether the predictive effects of the visual novelty detection task are stable across language development. Our first aim was therefore to explore this in a systematic manner. We conducted a longitudinal study in which visual novelty detection was measured at 9 months (together with perceptual tasks, not reported here), while repeated measures of infants’ vocabulary (number of words produced and comprehended) were taken at 12, 14, 18, and 24 months. The longitudinal design was similar to that previously used by Benasich and Tallal (1996, 2002). Second, if typical and atypical participants differed in their novelty detection ability, typical infants may also exhibit relevant individual differences. We predict that typical infants at least as a group will show a significant novelty preference in this relatively simple habituation-dishabituation task. Weaker ability to disengage attention from a familiar stimulus and/or poorer ability to encode, store and retrieve relevant information might impact language learning in the typical population, as well, even if the outcomes remain within the normal range (e.g., Rose et al., 2009). In the present study, we thus tested a group of typical participants in order to explore individual differences within typical development. An individual analysis of the linguistic outcomes was performed looking for a possible relation with the novelty detection task. Third, our sample consisted of French-learning infants, while previous studies tested English-learning infants. Since the trajectory of vocabulary growth may differ as a function of differences between the grammatical structures of languages (e.g., Slobin and Bever, 1982; Floccia et al., 2018), it is relevant to test a variety of languages.

## Materials and Methods

As part of a larger longitudinal study, at the first visit to the laboratory at 9 months, each infant was tested on the visual novelty detection task in addition to other tasks not reported here. We indeed aimed to perform a simple visual task, since infants were first tested with a complex task, the RAP task, to evaluate specific acoustic abilities. This procedure is particularly difficult and demanding for young participants. All infants were followed longitudinally and parents were asked to complete the online version of the French adaptation of the CDI (MacArthur Communicative Development Inventory, IFDC French version 1999) at 12, 14, 18, and 24 months of age.

### Participants

This study was carried out in accordance with the recommendations of CER-Paris Descartes (the ethics committee of the Université Paris Descartes), ethics approval nr 2016/32. All parents of all participating infants gave written informed consent prior to participation in accordance with the Declaration of Helsinki.

Forty-six 9-month-olds (19 girls, age range: 8–10 months) were included in the original cohort and tested on the RAP task. We selected this age range since the original design also included an acoustic task for which it was not possible to test participants earlier. Moreover, since 9 months is the age at which the first signs of word learning are observed, it was important to investigate the response to novelty at the beginning of this process. All infants were born full-term, had no history of hearing, language, or visual impairments, no recent occurrences of ear infections and no family history of congenital hearing loss. French was the only language spoken in the families. Among these 46 infants, fourteen failed to complete the RAP task, and were thus excluded from the longitudinal study. The remaining 32 participants (12 girls, mean age: 9 months and 1 day, age range: 8–10 months, *M* = 9.17 months, *SD* = 21.73 days) were administered the novelty detection task and were followed longitudinally by means of the CDIs. The number of infants whose parents successfully completed the CDI over time is presented in Table 1.

**Table 1:** *n* of participants included over time.

	Novelty detection (9 months)	CDI 12 months	CDI 14 months	CDI 18 months	CDI 24 months
Total *n* of participants	32 (*F* = 12)	28 (*F* = 10)	27 (*F* = 10)	21 (*F* = 8)	12 (*F* = 3)


### Habituation/Visual Novelty Detection Task

#### Stimuli

The experimental session started with a picture of a black and white checkerboard (23 × 52 cm, 652 × 1474 pixel, 72 dpi) used as pre-test image. The pre-test image was used to center infants’ attention to the screen before the presentation of each test trial. Infants were habituated to one adult female face with a neutral expression (size on the screen: 23 × 52 cm, 652 × 1474 pixel, 72 dpi). The face was presented in two identical copies side by side on a computer screen. In the test, immediately following habituation, one copy (left or right) of the familiarized female face was replaced by a novel face, that of a male child. At the beginning of each habituation and test trial, an attention getter of a colored turtle was presented in order to attract the infants’ attention to the screen. The experiment was run with Habit 2.1 on a Mac with OS X, version 10.10.5.

#### Procedure

Infants performed the task in a sound-attenuated, dimmed testing room where a central television monitor was placed. Infants were seated on a caregiver’s lap, on a chair in the middle of the booth (75 cm away from the central screen). The caregiver listened to masking music in order to avoid influencing the infant’s response. Moreover, they were asked to close their eyes and to not interact with the infant during the task. A video camera placed above the central screen recorded the session and transmitted information, through a monitor, to the experimenter, placed outside the booth and thus blind to the experiment. The study started with the pre-test trial. The pre-test trial lasted 18 seconds or until the infant looked away for more than 2 s. The purpose of the pre-test trial was to help infants get comfortable with the setup and to establish a basic criterion for overall attention. Infants with very low looking times (below 4 s) would have been excluded from data analysis, but this exclusion criterion did not apply to any infant in our sample.

##### Habituation phase

During habituation the female adult face appeared simultaneously on the left and the right side of the screen in each trial. The minimum looking time was set to 0.3 s (based on Benasich and Tallal, 1996). Looks below this threshold were ignored by the software. Trials ended when a look away larger than 2 s occurred. The baseline (100%) looking time was defined as the mean looking time in the first two trials. The same stimuli were repeatedly presented until the habituation criterion was reached. The criterion was set to a looking time equal to or less than 50% of the baseline (mean of the first two trials).

##### Test phase

**FIGURE 1:**
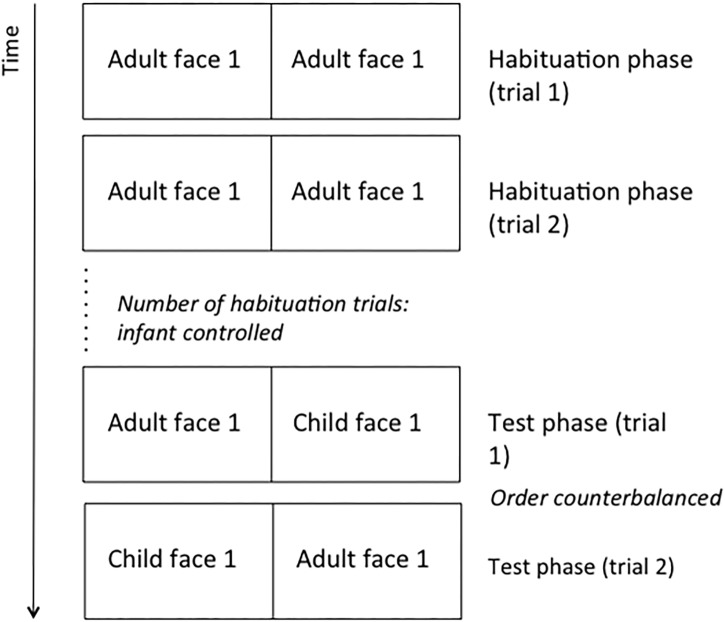
Schematic illustration of the experimental procedure.

During the test phase, the same adult face coupled with the face of a young child was used in the test phase as the novel stimulus. There were two test trials, and the child face appeared on the left and the right side of the screen, respectively, with the order counterbalanced across participants (for half of the infants, right first, left second, and vice versa for the other half of the infants Figure 1). Trials ended when a look away larger than 2 s occurred. The session was videotaped and looking patterns were scored online.

### Data Analysis

Several measures were obtained for each infant on the basis of the online data provided by the experimental software.

The habituation measures included (following Benasich and Tallal, 1996):

(1) The total looking time calculated as the cumulative time (in seconds) that each infant spent looking at the screen (pre-test + habituation phase + test phase)
(2) Looking time during the first habituation trial calculated as the looking time (in seconds) during the first habituation trial
(3) The number of habituation trials to criterion (TTC)
(4) Amount of response decrement (in %) calculated as: [(A–B)/A] × 100]; where A represents the mean of the first two habituation trials and B the mean of the last two habituation trials
(5) The linear regression slope (coefficient a) of each infant’s looking time across habituation trials

The novelty detection measure included (following Benasich and Tallal, 1996):

(6) Novelty effect (in %), calculated as: [N/(F+N) × 100]; where *N* represents the average looking time for the two test trials (novel items) and F the average looking time for the last two trials of the habituation.

### Standardized Questionnaires of Vocabulary Development

Infants’ language and cognitive abilities were assessed using the CDI at 12, 14, 18, and 24 months. At 12 and 14 months, parents completed the French adaptation of the long online CDI version: “Words and Gestures” (IFDC version 1999), whereas at 18 and 24 months, the French versions of the Hopkins “CDI: Words and Sentences” and the Hopkins “CDI: Phrases” were completed. For both comprehension questionnaires, we only compared the number of words comprehended, i.e., for the Words & Gestures form, we did not take into account the Gestures score, and for the Words & Sentences form we did not take into account the Sentences score.

Importantly, the CDI provides gender/age normed language scores assigning infants to percentile ranks ranging from 5 to 99. Crucially, however, as the current study aimed to investigate individual differences, the standardizing procedure was not performed, as it would have caused a significant loss of informative individual data. Thus, the raw score was used to define two dependent variables: the total number of words comprehended (receptive vocabulary) and the total number of words produced by each infant (productive vocabulary).

## Results

### Gender and Age

The means and standard deviations of the obtained measures are presented in Table 2. None of the reported measures showed a significant effect of gender between males (*n* = 12) and females (*n* = 16) in this sample, thus this variable was not analyzed further. Additionally, as the sample’s age ranged between 8 and 10 months, Pearson’s correlation was calculated between the each of the six measures and age (in days) at test. It suggested no significant effects (total looking time: *r* = -0.24; looking time during the first habituation trial: *r* = 0.09; the linear regression slope (coefficient a): *r* = 0.10; % novelty: *r* = -0.13; TTC: *r* = -0.15; % response decrement: -0.17).

**Table 2:** Means and Standard Deviations of the variables measured in the Habituation/visual novelty detection task.

Habituation/visual novelty detection variables	Mean	*SD*
First looking length (s)	12.8	7.4
Total looking time (s)	79.4	2.6
TTC	5.7	2.6
Habituation slope (α)	–0.3	0.3
% Novelty effect	66.7	12
% Response Decrement	35.2	35.8


### Novelty Preference

**FIGURE 2:**
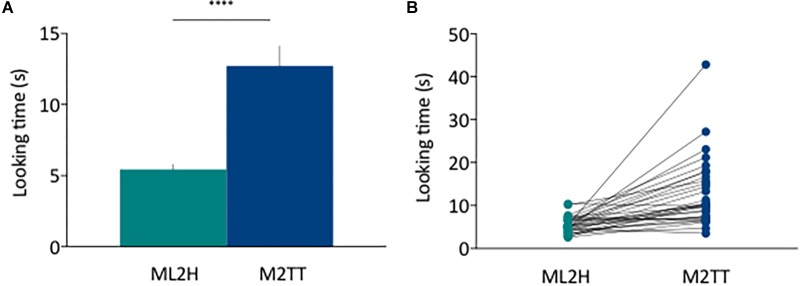
**(A)** Mean looking time of the two last habituation trials (ML2H) and the two trials during the test phase (M2TT) at the group level. **(B)** Plot of the individual variability of looking time between ML2H and M2TT. The Y-axis shows the looking time in seconds. Error bars represent the s.e. of the mean.

Infants’ novelty effect (in %), used as a measure of visual recognition memory, significantly exceeded chance (50%) (*M* = 66.7, *SD* = 12; *t* (31) = 7.86, *p* < 0.0001; *d* = 1.96), suggesting that as a group, infants successfully recognized the novel face. The raw looking time data is reported in Figure 2 showing a significant difference between the mean looking time of the two last habituation trials (ML2H) and the two trials during the test phase (M2TT): ML2H = 5.45 s, *SD* = 1.89; M2TT = 12.72; *SD* = 7.09; *t* (31) = 5. 27, ^∗∗∗∗^*p* < 0.0001; *d* = 1.24; power (1-β) = 0.99 both at the group (Figure 2A) and individual level (Figure 2B).

### Vocabulary Scores and Their Relation to Novelty Preference

**FIGURE 3:**
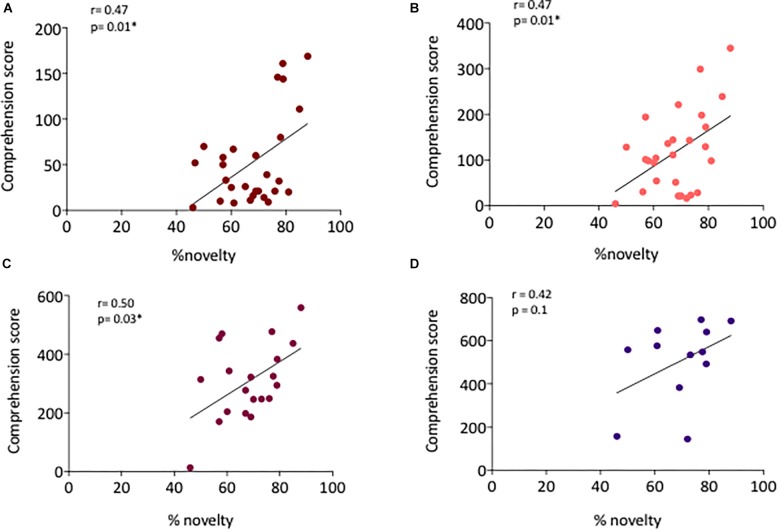
**(A)** Correlation between the % of novelty effect and the comprehension score at 12 months (*n* = 28), **(B)** 14 months (*n* = 27), **(C)** 18 months (*n* = 21), and **(D)** 24 months (*n* = 12).

At 12 months, vocabulary size ranged between 3 and 169 words comprehended and 0 and 18 words produced. Pearson’s correlation was positive and significant between the comprehension score and the % of novelty effect (*r* = 0.47, *p* = 0.01, Figure 3A). No correlation with the production score was found, most likely due to a floor effect, as the production scores were very low at this age.

At 14 months, vocabulary size ranged between 4 and 345 words comprehended and between 0 and 81 words produced. The correlation between the % novelty effect and the comprehension score was significantly positive (*r* = 0.47, *p* = 0.01, Figure 3B). No correlation with the production score was found due to a possible floor effect.

At 18 months, vocabulary size ranged between 13 and 559 words comprehended and 0 and 236 words produced. Again, the correlation between the comprehension score and the % novelty was positive and significant (*r* = 0.50, *p* = 0.025, Figure 3C). Moreover, a positive tendency was also found with the production score and the % novelty effect (*r* = 0.31, *p* = 0.1).

At 24 months, vocabulary size ranged between 144 and 698 words comprehended and between 9 and 610 words produced. A positive, although non-significant correlation was founded between the % of novelty effect and the comprehension score (*r* = 0.42, *p* = 0.1, Figure 3D) as well as between the % of novelty effect and the production score (*r* = 0.28, *p* = 0.3). Likely, the small sample size (*n* = 12) is responsible for the lack of significance (power analysis for the sample size: (1-β) = 0.286).

**FIGURE 4:**
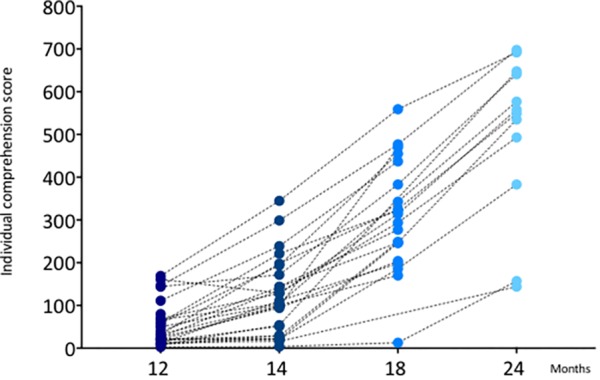
Infants’ receptive vocabulary scores between 12 and 24 months. Y-axis shows the individual raw score, the X-axis shows the ages in months.

Importantly, Pearson’s correlations across measures of vocabulary were also calculated, revealing a strong pattern of correlation between all measures of the receptive (Table 3) and expressive (Table 4) vocabulary across ages. Figure 4 shows the developmental trajectory of infant’s receptive vocabulary between 12 and 24 months.

**Table 3:** Correlations between the % novelty effect and the comprehension score and between the repeated measures of language outcomes themselves.

Comprehension	12 months	14 months	18 months	24 months
Novelty %	0.478*	0.467*	0.497^∗^	0.423
12 months				0.601^∗^
14 months	0.770**			0.749^∗^
18 months	0.669*	0.789*		0.954^∗∗^


*^∗^Correlation is significant at the 0.05 level (2-tailed). ^∗∗^Correlation is significant at the 0.01 level (2-tailed).*

**Table 4:** Correlations between the % novelty effect and the production score and between the repeated measures of language outcomes themselves.

Production	12 months	14 months	18 months	24 months
Novelty %	0.323	0.355	0.310	0.279
12 months				0.759^∗∗^
14 months	0.810**			0.604^∗^
18 months	0.668**	0.577**		0.822^∗∗^


*^∗^Correlation is significant at the 0.05 level (2-tailed). ^∗∗^Correlation is significant at the 0.01 level (2-tailed).*

For the summary of non-significant correlations, see Table 5. The positive correlations found between the novelty effect and vocabulary size at different ages implies that infants who are better at recognizing novelty later develop a larger vocabulary size. Importantly, this pattern of results was robust and similar across ages, suggesting that the % novelty is a stable predictor of linguistic abilities over time. This is in line with previous evidence that linked the ability to process novel information with later cognitive behavior and memory skills (e.g., Colombo et al., 1989) as well as with language vocabulary (e.g., Thompson et al., 1991; Rose et al., 2009).

**Table 5:** Correlations between the variables measured in the Habitation/visual novelty detection task and the comprehension scores measured across ages.

Habituation variables	TTC	First looking length (s)	% Response decrement	Total looking time (s)	Habituation slope (α)
**Comprehension scores**					
12 months	0.414*	–0.298	0.154	0.290	–0.464*
14 months	0.241	–0.310	0.087	0.242	0.340
18 months	0.302	–0.307	0.209	0.194	–0.268
24 months	0.158	–0.294	0.382	–0.222	–0.474


*^∗^Correlation is significant at the 0.05 level (2-tailed).*

In addition, a series of stepwise linear regressions were run by entering, as independent predictors, both the habituation variables and the infants’ age at the day of test, simultaneously. As a result, none of the variables entered in the models constantly, confirming that the % novelty was the only best predictor found across ages.

## Discussion

The present study sought to evaluate whether general cognitive skills measured using a visual novelty detection task is predictive of a language measure at 12, 14, 18, and 24 months. We aimed to test whether a measure of cognitive abilities, the novelty detection, is a stable predictor of linguistic outcomes across time. We reported evidence about the link between early response to novelty and the linguistic abilities over time. To our knowledge, no previous studies in the literature have shown such a strong and stable relationship between these variables in the same cohort of infants across time. Here, the % novelty effect was significantly correlated with the comprehension scores at 12, 14, and 18 months. Moreover, a positive tendency was found with the comprehension score at 24 months as well as with the scores in production. The existence of correlations between general cognitive skills and later language outcomes implies that, by combining linguistic and cognitive measures, a more subtle understanding of individual language development may be obtained. This shows that in addition to language-specific processes, assessing language-external factors informatively contributes to language acquisition studies, especially those interested in individual variation.

Our results are in line with previous findings (Thompson et al., 1991; Rose et al., 2009) showing the predictiveness of novelty preferences on language outcomes. Importantly, only one of the habituation variables, the slope, was predictive of later vocabulary in our study. The other habituation measures did not show a correlation with vocabulary, suggesting that habituation is not a strong predictor. More generally, therefore, it is not the ability to disengage from the familiar stimuli that is relevant for vocabulary learning, but rather the ability to notice new ones. Generally, infants who have better cognitive skills are more likely to have memory traces or processing abilities that are highly stable, increasing the probability of better performance. On the other hand, infants with limitations in recognition and retention are more likely to need more repetitions or more time in order to retrieve the same information (see Rose et al., 2009 for a review). Further work is needed to better understand exactly what attentional or processing mechanisms allow some infants to better notice and respond to novelty.

Our results with typical infants, even if not conclusive, suggest long-term predictability of language growth of the basis of individual abilities in the visual modality. Specifically, we suggest that general cognitive measures and, in particular, the % of novelty effect, have predictive value on later language abilities.

These relevant findings notwithstanding, some limitations of the study need to be discussed here. First longitudinal studies provide correlational, but not causal information. Hence, it might be the case that novelty preference and vocabulary size are correlated but not causally related, as they may both depend on another variable, not measured in a given study. Correlational results, in general, thus need to be interpreted carefully.

Second and relatedly, in longitudinal studies, the drop-out rate over time is often important. For this reason, CDI data could not be obtained for all infants originally tested at 9 months in the novelty detection task. The smaller number of participants, particularly at 24 months, lowered the statistical power of the correlations.

Third, we used faces as visual stimuli following Benasich and Tallal’s (1996) original study. While the visual stimuli were faces, our study did not necessarily require sophisticated face recognition abilities, as the adult female face and the child face differed in many basic visual features (e.g., shape and size of face, etc.). This was not a problem, as our study intended to assess general attentional and cognitive abilities, and not face perception *per se*. We note, however, that more sophisticated facial recognition abilities may be face-specific and independent of language, as one study found poor correlations between face recognition skills and visual and verbal recognition scores (Wilmer et al., 2010). Future studies will need to test to whether the results observed here were specific to the face-domain, or whether the same predictive power may be achieved using different visual stimuli.

In addition, not only basic visual features, but also gender of the face can affect infants’ ability to recognize novel faces. In particular, the gender of the main caregiver seems to significantly affect both spontaneous and novelty preferences (Quinn et al., 2002).

In our sample, 78.5% of the infants had a female primary caregiver. This specific information was collected through a questionnaire filled out by the parents before each test session. Since the majority of the babies were more often exposed to a female than to a male caregiver, we chose to use female faces for the habituation stimuli and male faces for the test stimuli. Despite any potential *a priori* preference for female faces, infants in this study exhibited a strong novelty effect for the male child face presented during the test as the novel stimulus, strengthening our result.

## Conclusion

This study contributes to a better understanding of whether cognitive measures evaluated at early stages are useful predictors of later language outcomes. Specifically, the novelty effect was found to be an early predictor of later vocabulary size between 12 and 24 months. Our findings imply that, by combining linguistic and cognitive measures, a more subtle understanding of individual language development may be obtained.

## Author Contributions

CM and JG developed the study concept. Testing, data collection, analysis, and interpretation were performed by CM under the supervision of JG. CM drafted the manuscript. JG provided critical revisions. All authors contributed to the study design and approved the final version of the manuscript for submission.

## Conflict of Interest Statement

The authors declare that the research was conducted in the absence of any commercial or financial relationships that could be construed as a potential conflict of interest.
